# Occurrence of extended-spectrum beta-lactamase (ESBL) in Gram-negative bacterial isolates from high vaginal swabs in a teaching hospital in Nigeria

**DOI:** 10.4314/gmj.v58i4.7

**Published:** 2024-12

**Authors:** Oluwatoyin B Famojuro, Tayo I Famojuro, Oluremi B Oluwatobi, Damilare D Olumide

**Affiliations:** 1 Department of Pharmaceutical Microbiology, Faculty of Pharmacy, Olabisi Onabanjo University, Sagamu, Ogun state, Nigeria; 2 Department of Pharmacognosy, Faculty of Pharmaceutical Sciences, Bingham University, Karu, Nasarawa State, Nigeria; 3 Department of Pharmaceutical Microbiology, Faculty of Pharmacy, University of Ibadan, Ibadan, Nigeria

**Keywords:** High vaginal swab, Antibiotic resistance, Gram-positive bacteria, Gram-negative bacteria, Extended-spectrum beta-lactamase

## Abstract

**Objective:**

This study aims to determine the antibiotic susceptibility pattern and incidence of extended-spectrum beta-lactamase (ESBL) genes in isolates from vaginal discharge of symptomatic female patients.

**Study design:**

Cross-sectional study

**Participant:**

Pregnant and non-pregnant women between 18 and 50 years who presented with genital tract infection and had not received antimicrobial therapy in the two weeks prior

**Interventions:**

The study determines the prevalence of bacteria in the vaginal discharge of female patients of reproductive age, the antibiotic susceptibility pattern of the isolates and the incidence of ESBL genes in Gram-negative isolates from the sample.

**Results:**

Bacteria were found in 74 (80.4%) and 88 (81.5%) samples from pregnant and non-pregnant women, respectively. *Escherichia coli* (n=48; 27.6%) occurred mostly in the samples, followed by *Staphylococcus aureus* (n=38; 21.8%). Among the Gram-positive, all *Streptococcus. pneumoniae* and *Staphylococcus. epidermidis* were sensitive to imipenem and meropenem (100%). *S. aureus* was the most resistant to cephalexin (71.4%), cefoxitin (60.5%) carbenicillin (60.5%) and ceftazidime (57.9%). *Escherichia coli* was highly resistant to carbenicillin (85.4%), cephalexin (64.6%) and cefotaxime (56.3%). *Klebsiella pneumoniae* showed the highest level of imipenem resistance (31.6%), followed by *E. coli* (29.2%). The prevalence of ESBL genes in Gram-negative isolates from pregnant women was 25.6% (11/43), compared to 30.3% (23/76) in non-pregnant women. Both *bla*_TEM_ and *bla*_SHV_ had the highest occurrence of 14.3% (17/119) of the isolates.

**Conclusion:**

This study found Gram-negative pathogens isolated from the vaginal tract of both pregnant and non-pregnant women to be resistant to multiple antibiotics and have ESBL genes.

**Funding:**

None declared

## Introduction

The vaginal flora is a complex environment with communities of microbes in different numbers and proportions, and it is predominantly populated with *Lactobacillus* species, helping maintain the vaginal pH acidic and constant.^1^,^2^ Lactobacilli that originate in the vagina survive for as long as the pH remains acidic. When the pH of the environment becomes neutral, a mixed flora of cocci and bacilli forms and persists until adolescence. Aerobic and anaerobic lactobacilli multiply during adolescence and serve to maintain acid pH by producing acid from glycogen, which appears to be an important strategy for hindering the proliferation of potentially dangerous bacteria. Bacterial vaginosis can occur when antimicrobial medicines suppress lactobacilli.^3^ Bacterial vaginitis (BV) is a genital infection caused by an overgrowth of bacteria in the vagina and characterised by vaginal discharge, irritation, and a foul odour that may be transitory or persistent.^4^-^6^

The alteration in vaginal ecology normally results in a decrease in the proportion of H_2_O_2_-generating bacteria, speeding up the colonisation of other species such as *Gardnerella vaginalis Mobiluncus* species, *Peptococcus* species, Bacteroides species and *Mycoplasma hominis*.^7^ Since BV is a frequent vaginal illness in women of reproductive age, interest in the condition has grown recently due to mounting evidence of its harmful consequences, including amniotic fluid infections, clinical chorioamnionitis, preterm labour, pelvic inflammatory disease and postpartum endometritis.^8^ One of the most frequent causes of reproductive tract infection (RTI) is bacterial vaginosis, and many factors, including douching, regular use of soap or herbal remedies, sexual activity, vaginal hygiene habits, and having several sex partners HIV infection, marital status, and sexually transmitted infections (STIs) has been linked to the prevalence of this condition.^9^,^10^ Some studies have linked sexually transmitted diseases such as syphilis, gonorrhoea, trichomoniasis, and chlamydial infections with preterm labour.^3^

Extended-spectrum beta-lactamases (ESBLs) can break down broad-spectrum cephalosporins and monobactams, as well as co-resistance against several different categories of antibiotics such as quinolones, aminoglycosides and cotrimoxazole.^11^ Resistance to routinely prescribed medications by extended-spectrum beta-lactamase-producing Gram-negative bacteria is rising.^12^ It has been found that Gram-negative bacteria isolated from the vaginal have extended-spectrum beta-lactamase.^12^-^14^ ESBL-producing Gram-negative bacteria could expose women to infections of the reproductive system and increase the likelihood of newborn mortality during pregnancy.^12^

ESBL-producing Gram-negative bacteria are increasingly resistant to antibiotics that are frequently used.^12^ The rising incidence of ESBL-producing Gram-negative bacterial infections in the last few years has become a global issue, leaving patients with few therapeutic options.^15^ There are numerous reports on ESBL-producing bacteria causing infections in humans in Nigeria, 16-18.

Still, there are few reports on the characterisation of ESBL genes in isolates from pregnant and non-pregnant women. Therefore, this study was conducted to identify ESBL-producing Gram-negative bacteria isolated from the vaginal of pregnant and non-pregnant women with genital tract infections.

## Methods

### Study site and sample collection

Two hundred (200) high vaginal swabs were collected from the Medical Microbiology Laboratory of Olabisi Onabanjo University Teaching Hospital between January and October 2023.

The samples were from both pregnant and non-pregnant women between the ages of 18 to 50 years diagnosed with genital tract infections.

### Isolation and identification of bacterial isolates

The swabs were inoculated on mannitol salt agar's and MacConkey agar's surface and incubated for 24 hours. Colonies on MacConkey agar were further subcultured onto Eosin methylene blue agar, cetrimide agar and deoxycholate citrate agar. The isolates were then Gram stained and further identified with biochemical tests such as citrate, indole, methyl red, Voges-Proskauer, hydrogen sulphide production, catalase, coagulase, sugar fermentation and gas production.^19^

### Antibiotic susceptibility testing

The antibiotic susceptibility profile of the bacteria was determined with the Kirby-Bauer disk diffusion test.^20^ For Gram-negative isolates, imipenem (10µg), aztreonam (30µg), meropenem (10µg), cefoxitin (30µg), cephalexin (30µg), amoxicillin/clavulanic acid (20µg/10µg), ciprofloxacin (5µg), amikacin(10µg), carbenicillin (30µg), cefotaxime (30µg), ceftazidime (30µg) and cefepime (30µg) were used. For Gram-positive isolates, imipenem (10µg), meropenem (10µg), cefoxitin (30µg), cephalexin (30µg), amoxicillin/clavulanic acid (20µg/10µg), ciprofloxacin (5µg), amikacin(10µg), carbenicillin (30µg), cefotaxime (30µg), ceftazidime (30µg) and azithromycin (15µg) were utilized.

Briefly, isolates were subcultured on Nutrient agar plates and incubated for 18 hours. Then, 3-4 colonies were picked and suspended in sterile distilled water, and the turbidity was adjusted to 0.5 McFarland standard. Sterilised molten Mueller Hinton agar cooled to about 45 °C was poured into sterile Petri dishes and solidified at room temperature. The surface of the plates was dried in an oven at 37 °C. A sterile swab stick was then dipped in the McFarland suspension and used to inoculate the Mueller-Hinton agar plates. A sterile pair of forceps was used to place the antibiotic discs on the surface of the Mueller-Hinton agar plate. The plates were left on the bench for 1 hour and then incubated at 37 °C for 18 hours. The zones of growth inhibition were measured and recorded in millimetres. According to Clinical Laboratory and Standard Institutes performance standards for antimicrobial susceptibility testing, the result was interpreted as sensitive, resistant, or intermediate.^21^

### Phenotypic ESBL detection

ESBL production was detected with a modified double disk synergy test. Amoxicillin/clavulanic acid (20µg/10µg) was placed at the centre of freshly inoculated Mueller-Hinton agar plate, ceftazidime (CAZ; 30 µg), cefotaxime (CTX; 30 µg) and cefepime (CFP, 30 µg) were placed 20mm apart from the amoxicillin/clavulanic acid disc with the aid of a sterile pair of forceps. The plates were left on the bench for 1 hour and Then incubated at 37°C for 18 hours. When the inhibition zone around any cephalosporin expanded toward the central disc with amoxicillin/clavulanic acid, the sample was classified as positive for ESBL.^22^

### DNA extraction

The DNA of the bacteria was extracted using the boiling process. A pure colony of each isolate was inoculated in 2 millilitres of Luria Bertani broth (LB), cultured for 18 to 24 hours, and centrifuged for 10 minutes at 10,000 rotations per minute. To release the DNA, the bacterial cells were submerged in 500 µL of phosphate buffer (100 mM, pH 7), and then they were heated to 100 °C in a boiling water bath for 15 minutes to weaken their membranes. Then, 250 µL of absolute alcohol was used to precipitate the DNA, which was then centrifuged one more and resuspended in 100 µL of sterile water.^23^

### Molecular detection of ESBL

The thermal cycler (Applied Biosystems, USA) was used to perform PCR to determine the presence of the *bla*_TEM_, *bla*_SHV_ and *bla*_CTX-M_ -ESBL genes. The primer sequences utilised are listed in Table 1. One microliter of DNA, 1 µL of each primer (0.2 pmol/µl) from Inquaba, South Africa, 10 µL of WizPure^TM^ PCR 2X Master mix from Wizbiosolutions, South Korea, and 7 µL of protease/DNase/RNase-free water made up the 20 µL PCR final volume. The amplification requirement is a 10-minute initial denaturation step at 94 °C. 30 cycles of denaturation (40 s at 94 °C), annealing (40 s at 60 °C), and elongation (one minute at 72 °C), followed by a final elongation step (seven minutes at 72 °C). To view the PCR products, a 1.5% agarose gel stained with ethidium bromide was electrophoretically migrated at 100 volts for an hour. A marker with 100 bp was used as a comparison. Following migration, the various bands were examined under UV light. After migration, the bands were visible under UV light.^24^,^25^

**Table 1:** Primer sequences of ESBL genes

Genes	Primer sequence	Amplicon sizes (bp)	Reference
SHV	F-5′TCGCCTGTGTATTATCTCCC-3′ R-5′ CGCAGA-TAAATCAC-CACAATG-3′	768	Maynard *et al.* 24
TEM	F-5′GAG-TATTCAACATTTTCGT-3′ R-5′ AC-CAATGCTTAATCAGTGA-3′	857	Maynard *et al.* 24
CTX-M	F-5′TTT-GCGATGTG-CAGTACCAG-TAA-3′ R-5′CGATAC-GTT-GGTGGTGCCATA-3′	544	Edelstein *et al.*^25^

### Statistical analysis

Fischer exact test was used to determine the statistically significant difference in the presence of bacteria in the HVS samples of pregnant and non-pregnant (*p* = 0.05)

### Ethical clearance

Ethical approval was obtained from the Health Research Ethics Committee of the Olabisi Onabanjo University Teaching Hospital, Sagamu, Ogun state (OOUTH/HREC/590/2023AP)

## Results

### Patient demographic information, Isolation and

A total of 200 samples were collected from 92 pregnant and 108 non-pregnant women between the ages of 18 to 50 years. Out of 200 HVS samples, 162 (81.0%) were positive for bacterial isolates. Gram-positive bacteria were isolated in 43 of the HVS samples, 107 samples yielded Gram-negative isolates while 12 samples yielded both Gram-positive and Gram-negative isolates. Bacteria were found in 74 (80.4%) and 88 (81.5%) samples from pregnant and non-pregnant women, respectively. Bacterial isolates were more prevalent (84.8%) in samples from pregnant women aged 31-40, while the largest proportion of bacteria (87.5%) was identified in non-pregnant women aged 21-30 (Table 2).

**Table 2:** Percentage of pregnant and non-pregnant women with bacterial-positive samples with age

Ages in year	Pregnant (n = 92)		Non-pregnant (n = 108)	
	Number of samples	No of positive samples (%)	Number of samples	No of positive samples (%)
**18 - 20**	7	5 (71.4)	8	4 (50.0)
**21 – 30**	28	23 (82.1)	24	21 (87.5)
**31 – 40**	46	39 (84.8)	53	45 (84.9)
**41 – 50**	11	7 (63.6)	23	18 (78.3)
Total	**92**	**74 (80.4)**	**108**	**88 (81.5)**

### Prevalence bacterial isolates in HVS samples

**Figure 1:**
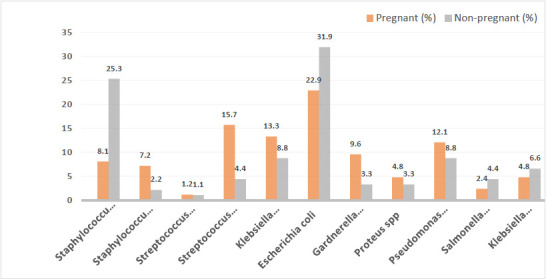
Prevalence of bacterial isolates in HVS samples from pregnant non-pregnant women

A total of 174 isolates, including 55 Gram-positive isolates and 119 Gram-negative isolates, which consist of *Staphylococcus aureus, Staphylococcus epidermidis, Streptococcus pneumoniae, Klebsiella pneumoniae, Escherichia coli, Enterobacter* spp., *Proteus* spp., *Pseudomonas aeruginosa, Salmonella* spp., *Klebsiella oxytoca. Escherichia coli* (n=48; 27.6%) occurred mostly in the samples, followed by *Staphylococcus aureus* (n=38; 21.8%) (Table 3). *S. aureus* (25.3%), *E. coli* (22.9%), *Salmonella* species (4.4%) and *K. oxytoca* (6.6%) were more prevalent in HVS samples of non-pregnant than pregnant women (Figure 1). The presence of bacteria in the HVS samples of pregnant and non-pregnant women was not statistically significant (*p* = 0.859).

**Table 3:** Percentage of bacterial isolates found in the HVS sample

Bacterial isolates	Number (%)
***Staphylococcus aureus***	38(21.8)
***Staphylococcus epidermidis***	10(4.6)
***Streptococcus pneumoniae***	7(4.0)
***Klebsiella pneumoniae***	19(10.9)
***Enterobacter* spp.**	11(6.3)
***Proteus* spp.**	7(4.0)
***Pseudomonas aeruginosa***	18(10.4)
***Salmonella* spp.**	6(3.5)
Total	**174(100)**

### Antibiotic susceptibility profile of the isolates

**Figure 2:**
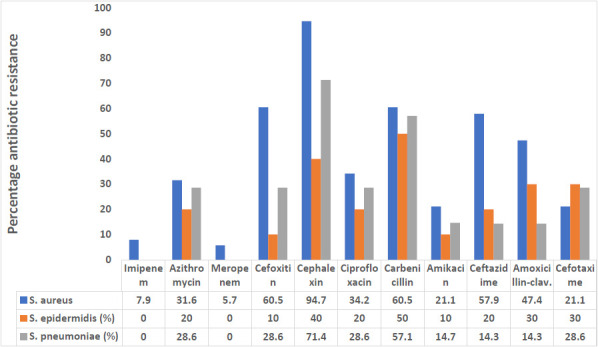
Antibiotic resistance patterns of Gram-positive bacterial isolates

**Figure 3:**
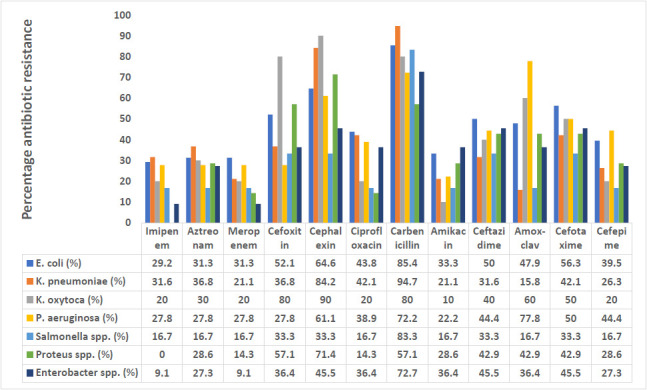
Antibiotic resistance profile of Gram-negative bacterial isolates

Among the Gram-positive, all *S. pneumoniae* and *S. epidermidis* were sensitive to imipenem and meropenem (100%), while a lower resistance rate to meropenem (5.7%) and imipenem (7.9%) was observed in *S. aureus*. *S. aureus* was the most resistant to cephalexin (71.4%), cefoxitin (60.5%), carbenicillin (60.5%) and ceftazidime 57.9% followed by *S. pneumoniae* (Figure 2). Among the Gram-negative bacterial isolates, *E. coli* was highly resistant to carbenicillin (85.4%), cephalexin 64.6% cefotaxime (56.3%). *K. pneumoniae* was the most resistant to carbenicillin (94.7%), followed by *Salmonella* species (83.3%), while *K. oxytoca* was the most resistant to cephalexin (90%) and cefoxitin (80%). *K. pneumoniae* showed the highest level of imipenem resistance (31.6%), followed by *E. coli* (29.2%). *P. aeruginosa* was the most resistant isolate to amoxicillin-clavulanic acid. A 71.4% and 57.1% resistance rate were observed for cephalexin and carbenicillin, respectively, among *Proteus* spp. (Figure 3).

### Detection of ESBL in Gram-negative isolates

ESBL genes were detected in 34 (28.6%) of the isolates including 31 phenotypically positive and three phenotypically negative isolates for ESBL, 18 *E. coli*, 7 *P. aeruginosa*, 5 *K. pneumoniae*, 3 Enterobacter spp. and 1 *K*. *oxytoca*. The overall prevalence of ESBL genes in Gramnegative isolates from pregnant women was 25.6% (11/43), compared to 30.3% (23/76) in non-pregnant women. Both *bla*_TEM_ and *bla*_SHV_ had the highest occurrence of 14.3% (17/119 while *bla*_CTX-M_ was found in 5.9% (7/119) of the isolates. Five (4.2%) of the isolates (including three *E. coli*, one *P. aeruginosa* and one *K. pneumoniae*) had both *bla*_TEM_ and *bla*_SHV_, whereas *bla*_SHV_ and *bla*_CTX-M_ occurred together in 1.7% (2/119) *E. coli* isolates (Table 4).

**Table 4:** Occurrence of extended-spectrum beta-lactamase (ESBL) in cephalosporin-resistant Gram-negative bacteria

ESBL-genes	Pregnant women (n = 43)	Non-pregnant women (n = 76)	Total (n = 119)
***bla***_**TEM**_	4 (28.6)	8 (26.7)	12 (10.1)
***bla***_**CTX-M**_	2 (21.4)	3 (10.0)	5 (4.2)
***bla***_**SHV**_	2 (14.3)	8 (26.7)	10 (8.4)
***bla***_**TEM**_ **and *bla***_**SHV**_	3 (14.3)	2 (6.7)	5 (4.2)
***bla***_**SHV**_ **and *bla***_**CTX-M**_	0 (0)	2 (6.7)	2 (1.7)
**Total**	11 (25.6)	23 (30.3)	34 (28.6)

## Discussion

Bacterial vaginosis (BV), the most prominent cause of abnormal vaginal discharge among women of reproductive age, has been linked with a considerable risk of morbidity in women.^26^ This study reported the presence of both Gram-positive and Gram-negative isolates in the vaginal discharge of pregnant and non-pregnant women of reproductive age. The isolates include *Staphylococcus aureus, Staphylococcus epidermidis, Streptococcus pneumoniae, Klebsiella pneumoniae, Escherichia coli, Enterobacter* species, *Proteus* species, *Pseudomonas aeruginosa, Salmonella* species and *Klebsiella oxytoca*. In this study, 68.4% of the bacteria were Gram-negative, while 55 (31.6%) were Gram-positive. Other research has also indicated a higher occurrence of Gram-negative bacteria.^27^,^28^ *E. coli* was the most isolated pathogen (27.6%), followed by *S. aureus* (21.8%) and *K. pneumoniae* (10.9%). Ravishankar and Prakash^28^ and Effiong *et al.*^29^ also reported *E. coli* as the most predominant isolate with 38.8% and 36.7% prevalence, respectively, while Ahabwe *et al.*^30^ documented that *S. aureus* (48.6%) and *K. pneumoniae* (29.9%) were the most frequently isolated bacteria. Contrary to a study by Mulu *et al.*^26^ that found bacterial vaginosis was more common in non-pregnant than pregnant women (*p* = 0.002), there was no statistically significant difference in the presence of bacterial pathogens -between pregnant (80.4%) and non-pregnant (81.5%) (*p* = 0.8586) symptomatic patients. While our study found a greater incidence of 80.4% of harmful bacteria in the vaginal discharge of pregnant women, Ravishankar and Prakash^28^ reported a 50.4% prevalence. This study found that pregnant women between the ages of 31 and 40 had the highest frequency of bacterial infection; in contrast, non-pregnant women between the ages of 21 and 30 had the highest frequency, while Narayana-Swamy *et al.*^31^ noted the maximum prevalence of bacterial vaginosis among women between the ages of 20 and 30 years.

Antibiotic resistance has recently gained attention in healthcare settings around the world due to its implications on rising healthcare expenditures, morbidity, and mortality among patients with infectious illnesses.^32^ In developing nations, information about antibiotic susceptibility patterns of bacterial isolates is often infrequent, exacerbating the problem.^32^,^33^ In this study, as Ravishankar and Prakash showed, lower resistance to imipenem was reported in Gram-positive (0 - 7.9%) than in Gramnegative (0 - 31.6%).^28^ *E. coli* showed the highest resistance to ciprofloxacin, ceftazidime, cefotaxime and meropenem. In contrast, Narayana-Swamy *et al.*^31^ reported lower resistance to imipenem and meropenem among *E. coli* isolates from the high vaginal swab. *K. pneumoniae, Salmonella* and *Proteus* species also showed lower resistance to ciprofloxacin and amikacin. Low resistance to ciprofloxacin by *Klebsiella* species was also reported.^28^

The *P. aeruginosa* isolates from vaginal discharge in the present study showed increased resistance to cephalosporins like ceftazidime (44.4%), cefotaxime (50.0%), cephalexin (61.1%), and cefepime (44.4%), however Effiong *et al.*^29^ observed reduced resistance. In this study, 60.5% of *S. aureus* were resistant to cefoxitin, indicating a high prevalence of methicillin-resistant *Staphylococcus aureus* (MRSA). The occurrence of MRSA (14.3%) among pregnant women with bacterial vaginitis has also been reported from the same region.^34^ These MRSA were also resistant to many antibiotics, including beta-lactams, necessitating special attention in bacteriologic studies of vaginal infections.^34^

Infections produced by ESBL-producing bacteria continue to present a problem in establishing the right treatment because they may be resistant to other classes of antibiotics.^12^,^35^ Neonatal sepsis has been reported in babies born by ESBL-infected mothers.^17^,^36^ This study reported the presence of ESBL in 28.6% of isolates from both pregnant and non-pregnant women, while Divya and Jayakumar^27^ reported a high incidence of 53%. According to this study, the most common gene was *bla*_TEM_, discovered in 10.1% of the isolates. *bla*_SHV_ was found in 8.4% of the isolates, while *bla*_CTX-M_ was identified in 4.2%. However, Ghaddar *et al.*^36^ found that among ESBL-producing *E. coli* from pregnant women's vaginal discharge, the most common gene was *bla*_CTX-M_ (90.7%), followed by *bla*_TEM_ (88.4%) and *bla*_SHV_ (44.2%). The prevalence of ESBL genes in Gram-negative isolates from pregnant women was 25.6%, lower than that of non-pregnant women (30.3%). However, a study from Ghana documented an ESBL prevalence rate of 41.4% among pregnant women.^37^

## Conclusion

This study found Gram-negative pathogens isolated from the vaginal tract of both pregnant and non-pregnant women to be resistant to multiple antibiotics and have ESBL genes. This study also reported the occurrence of methicillin-resistant *S. aureus* (MRSA), carbapenem-resistant *Pseudomonas aeruginosa* (CRPA) and carbapenem-resistant Enterobacteriaceae (CRE) that were resistant to multiple antibiotics. The presence of these MDR-resistant strains in the vagina might cause problems such as urinary tract infections and newborn meningitis that are difficult to treat. The outcome of this study may alert clinical microbiologists and healthcare providers about the existence of ESBL-producing organisms in the vagina. Therefore, an efficient antibacterial approach is required.
